# Lateral similarity and symmetry of biometric data from the LenStar in a population with cataract

**DOI:** 10.1371/journal.pone.0353664

**Published:** 2026-07-21

**Authors:** Achim Langenbucher, Jascha Wendelstein, Alan Cayless, Stefan Wagenpfeil, Peter Hoffmann, David Cooke, Nóra Szentmáry

**Affiliations:** 1 Department of Experimental Ophthalmology, Saarland University, Homburg/Saar, Germany; 2 Department of Ophthalmology, Ludwig Maximilians University Munich, Munich, Germany; 3 School of Physical Sciences, The Open University, Milton Keynes, United Kingdom; 4 Institute for Medical Biometry, Epidemiology and Medical Informatics, Saarland University, Homburg/Saar, Germany; 5 Augen- und Laserklinik Castrop-Rauxel, Castrop-Rauxel, Germany; 6 Great Lakes Eye Care, Saint Joseph, Michigan, United States of America; 7 Department of Neurology and Ophthalmology, Michigan State University, College of Osteopathic Medicine, East Lansing, Michigan, United States of America; 8 Department of Ophthalmology, Semmelweis-University, Budapest, Hungary; University of Warmia, POLAND

## Abstract

**Purpose:**

To investigate the similarity and symmetry of biometric measures between eyes in a large dataset of measurements of both eyes taken with the LenStar optical biometer.

**Methods:**

Cross-sectional non-randomised study evaluating a dataset containing 13,420 bilateral LenStar 900 biometric measurements on patients without history of eye surgery or ocular pathology taken before cataract surgery, consisting of scalar parameters axial length AL, corneal thickness CCT, anterior chamber depth ACD, lens thickness LT and corneal diameter WTW. The keratometric power vector components equivalent power KEQ were assessed for similarity, and the projections of corneal astigmatism vector KC0 and KC45 were analysed for direct and mirror symmetry with respect to the vertical (sagittal) plane.

**Results:**

Mean / standard deviation (squared correlation coefficient) difference between right and left eye measures was 0.02 ± 0.31(0.95) / 0.00 ± 0.01(0.93) / 0.00 ± 0.14(0.88) / −0.01 ± 0.22(0.78) / −0.02 ± 0.17(0.87) mm for AL / CCT / ACD / LT / WTW and −0.06 ± 0.48(0.92) dioptres for KEQ. Keratometric astigmatism showed a high degree of mirror symmetry (KC45 of left eyes inverted in sign) which outperforms direct symmetry. The median deviation of the keratometric axes of both eyes considering mirror symmetry was 15 degrees compared to 29 degrees for direct symmetry.

**Conclusions:**

In most cases, biometric measures match between eyes of a subject, but there are rare situations with large deviations between eyes. Keratometric astigmatism exhibits mirror symmetry. In most cases the biometric measures from the contralateral eye could be used where biometry is unavailable or to double-check before cataract surgery.

## Background

The invention of optical biometry with the launch of the first IOLMaster (Carl Zeiss Meditec AG, Jena, Germany) in 1999 was a milestone in modern cataract surgery. All relevant biometric parameters required for intraocular lens (IOL) power calculation could be measured in a single measurement procedure with one instrument. The repeatability and reproducibility of optical biometry outperform those of classical ultrasound biometry [1,2]. Together with modern IOL power calculation schemes the performance of optical biometry enabled the invention of some advanced IOL concepts such as multifocal, enhanced depth of focus, or monofocal plus IOLs.

Many studies have been performed in the past analysing the repeatability or reproducibility of optical biometers or comparing the biometric measures of various instruments [1,2]. These studies have shown that the precision of the biometric measures is systematically higher in optical biometry as compared to ultrasound, although the sets of measurements from different instruments are not fully interchangeable. A clear understanding of the uncertainties in the biometric measures and of the correlations between the uncertainties and potential heteroscedasticities would enable an error propagation model for predicting the resulting uncertainty in the IOL power or in the spectacle refraction after cataract surgery [3] to be established.

It is known that in most patients both eyes show similar biometric measures and refractions. Our study makes use of the lateral symmetry between the eyes – essentially that the left and right eyes are mirror images of one another. We would therefore expect many of the biometric measures of the eyes to exhibit the same mirror symmetry and for the measures from left and right eyes to be comparable given suitable adjustments (e.g., inversion of horizontal components) corresponding to reflection in the vertical (sagittal) plane. Since many of the corneal biometric parameters are essentially two-dimensional (e.g., astigmatism vectors or x-y coordinates of the pupil centre), where appropriate we will also refer to this as symmetry under reflection in the vertical axis for simplicity.

For scalar measures, this similarity between both eyes means that differences between left and right are quite low [4–8]. However, vector measures such as keratometry may exhibit either direct symmetry where all vector components of both eyes are similar in both sign and magnitude [9–13] or mirror symmetry [10], in which case the vector components would have to be preprocessed before testing for similarity [7]. In cases where optical biometry fails for one eye or is not available at the time of lens power calculation, these similarities and symmetries suggest that the corresponding measures from the contralateral eyes could be used to predict the IOL power [6,7,14,15]. However, we also know that in some patients the eyes are anisometric in one or more biometric parameters, and this could cause disparities of several dioptres (dpt) in the spectacle refraction or object-image magnification disparities in terms of aniseikonia [11,16,17].

Since ocular biometry is routinely measured in both eyes in many clinical centres, the comparison of the measures of both eyes could be used to double-check lens power prediction prior to cataract surgery and could potentially help to identify erroneous measurements [4,5,7,9].

The **purpose of the present study** was to analyse a large dataset of measurements from the LenStar optical biometer containing measurements of both eyes before cataract surgery

- to evaluate the differences between both eyes in biometric distances for relevant measures used for lens power calculation, and
- to analyse the inter-eye differences in keratometry (mean corneal power and keratometric astigmatism interpreted as a vector measure) in terms of direct symmetry and mirror symmetry with respect to the vertical axis.

## Methods

### Dataset for our evaluation

A large dataset containing N = 38,720 biometric measurements was considered in this study. All measurements were performed at the Great Lakes Eye Care Center (St. Joseph, Michigan, USA) with the LenStar 900 (Haag-Streit, Köniz, Switzerland) between March 2019 and March 2023. Measurements of eyes with any previous surgery or pathology (e.g., ectatic diseases such as keratoconus or pathologies such as scars) had already been excluded at the source. All procedures carried out in studies involving human participants were in accordance with the ethical standards of the Ärztekammer des Saarlandes and with the 1964 Helsinki declaration and its later amendments or comparable ethical standards. The local ethics committee (IRB) has provided a waiver for this study (Ärztekammer des Saarlandes, 157/21), since all data processed in this study were already anonymised at source before being transferred to us for processing. This precludes any back-tracing of the identity, and therefore informed consent of the patients was not necessary. This article does not contain any studies on animals performed by any of the authors.

The data were anonymised at source, stored as an .xlsx Excel data table using the software module for batch data export, and transferred to us for research purposes on 08/07/2024. Data tables were reduced to the relevant parameters required for our data analysis, consisting of the following measurements: patient ID, date of birth, examination date, the laterality (left or right eye), flat (K1) and steep (K2) keratometric power, both in dioptres, together with the flat axis Axis1 in degrees, axial length (AL) in mm, central corneal thickness (CCT) in mm, aqueous depth (AD) in mm (measured from corneal endothelium to the lens front apex), central thickness of the crystalline lens (LT) in mm, horizontal corneal diameter (WTW) in mm, pupil size (PD), the pupil barycentre defined as the chord between the coaxially sighted light reflex and the pupil centre in Cartesian coordinates [18,19] (PCX horizontally in mm, PCY vertically in mm), and the iris barycentre defined as the chord between the limbal centre and the coaxially sighted light reflex in Cartesian coordinates (ICX horizontally in mm, ICY vertically in mm) [18].

Incomplete datapoints were excluded and only bilateral measurements performed on the same examination day were qualified for the data processing. The data were transferred to Matlab (Matlab 2022b, MathWorks, Natick, USA) for further processing.

### Data pre-processing in Matlab

Each patient’s age (Age) in years was derived from the exam date and date of birth. From the corneal front surface data (K1, K2, Axis1) and the keratometer index used in the measurement (n_K_ = 1.3375), we extracted the corneal radii in the flat meridian R1 = 1000·(n_K_-1)/K1 and steep meridian R2 = 1000·(n_K_-1)/K2, both in mm, and the mean corneal radius R, calculated as the harmonic mean of R1 and R2 (R = 2·R1·R2/(R1 + R2)). Keratometric astigmatism was derived as KC = K2-K1. The standard notation of keratometry (K1, K2, Axis1) was converted to 3D power vector components with equivalent power defined as KEQ = 0.5·(K1 + K2) and the 2 astigmatic power vector components in terms of projections of keratometric astigmatism to the 0°/90° axis (KC0 = ½·KC·cos(2·Axis1)) and the 45°/135° axis (KC45 = ½·KC·sin(2·Axis1)). The phakic anterior chamber depth (ACD) was derived from CCT and AD as ACD = CCT + AD.

### Data processing in Matlab and statistics

For the three vectorial measures (keratometric astigmatism in either standard notation or component notation, the location of the Purkinje I image, and the location of the pupil centre) some additional parameters were defined (for left eyes only) in order to facilitate analysis of mirror symmetry with respect to the vertical axis [7]. Adopting the convention of mirroring the values from left eyes to correspond to those from right eyes, the respective parameters (indicated (.)M) read as follows:


$$\begin{array}{c} \left[\begin{array}{c} KC\text{0}M\\ KC\text{4}\text{5}M \end{array}]=[\begin{array}{c} KC\text{0}\\ -KC\text{4}\text{5} \end{array}\right] \end{array}$$

$$\begin{array}{c} \left[\begin{array}{c} ICXM\\ ICYM \end{array}]=[\begin{array}{c} -ICX\\ ICY \end{array}\right] \end{array}$$

$$\begin{array}{c} \left[\begin{array}{c} PCXM\\ PCYM \end{array}]=[\begin{array}{c} -PCX\\ PCY \end{array}\right] \end{array}$$

$$\begin{array}{c} Axis\text{1}M=\text{1}\text{8}\text{0}{\;}^{\circ }-Axis\text{1}\text{.} \end{array}$$

For each patient, the differences between the measures from the right eye (indicated by a subscript (.)_OD_) and the left eye (indicated by a subscript (.)_OS_) were calculated and marked with a Δ (biometric parameter of the right eye (.)_OD_ minus corresponding parameter of the left eye (.)_OS_). To test for direct symmetry (indicated with (.)D) we used the non-mirrored differences in the vector components


$$\begin{array}{c} \left[\begin{array}{c} \Delta KC\text{0}D\\ \Delta KC\text{4}\text{5}D \end{array}]=[\begin{array}{c} {KC\text{0}}_{OD}-{KC\text{0}}_{OS}\\ {KC\text{4}\text{5}}_{OD}-{KC\text{4}\text{5}}_{OS} \end{array}\right] \end{array}$$

$$\begin{array}{c} \left[\begin{array}{c} \Delta ICXD\\ \Delta ICYD \end{array}]=[\begin{array}{c} {ICX}_{OD}-{ICX}_{OS}\\ {ICY}_{OD}-{ICY}_{OS} \end{array}\right] \end{array}$$

$$\begin{array}{c} {}[\begin{array}{c} \Delta PCXD\\ \Delta PCYD \end{array}]=[\begin{array}{c} {PCX}_{OD}-{PCX}_{OS}\\ {PCY}_{OD}-P{CY}_{OS} \end{array}], \end{array}$$

and to test for mirror symmetry, we used the mirrored differences in the vector components


$$\begin{array}{c} \left[\begin{array}{c} \Delta KC\text{0}M\\ \Delta KC\text{4}\text{5}M \end{array}]=[\begin{array}{c} {KC\text{0}}_{OD}-{KC\text{0}M}_{OS}\\ {KC\text{4}\text{5}}_{OD}-{KC\text{4}\text{5}M}_{OS} \end{array}\right] \end{array}$$

$$\begin{array}{c} \left[\begin{array}{c} \Delta ICXM\\ \Delta ICYM \end{array}]=[\begin{array}{c} {ICX}_{OD}-{ICXM}_{OS}\\ {ICY}_{OD}-{ICYM}_{OS} \end{array}\right] \end{array}$$

$$\begin{array}{c} {}[\begin{array}{c} \Delta PCXM\\ \Delta PCYM \end{array}]=[\begin{array}{c} {PCX}_{OD}-{PCXM}_{OS}\\ {PCY}_{OD}-P{CYM}_{OS} \end{array}], \end{array}$$

For the keratometric axis, calculation of the differences between the right and left eye requires consideration of the periodicity with a period of 180 degrees (for keratometry) and 360 degrees (for the location of the Purkinje I image and the pupil centre). Therefore, the differences for direct symmetry (e.g., both eyes of a subject having a keratometer axis of Axis1_OS_ = Axis1_OD_ = 30°) were derived from


$$\begin{array}{c} \Delta Axis\text{1}D=min\{\begin{array}{c} \left|{Axis\text{1}}_{OD}-{Axis\text{1}}_{OS}\right|\\ \left|{Axis\text{1}}_{OD}-{Axis\text{1}}_{OS}-\text{1}\text{8}\text{0}{\;}^{\circ }\right|\\ \left|{Axis\text{1}}_{OD}-{Axis\text{1}}_{OS}+\text{1}\text{8}\text{0}{\;}^{\circ }\right| \end{array}\}\text{,} \end{array}$$

and the differences for mirror symmetry (e.g., for a situation where the left eye / right eye of a subject have axes of Axis1_OS_ = 20° / Axis1_OD_ = 160°) were derived from


$$\begin{array}{c} \Delta Axis\text{1}M=min\{\begin{array}{c} \left|{Axis\text{1}}_{OD}-{Axis\text{1}M}_{OS}\right|\\ \left|{Axis\text{1}}_{OD}-{Axis\text{1}M}_{OS}-\text{1}\text{8}\text{0}{\;}^{\circ }\right|\\ \left|{Axis\text{1}}_{OD}-{Axis\text{1}M}_{OS}+\text{1}\text{8}\text{0}{\;}^{\circ }\right| \end{array}\}\text{.} \end{array}$$

Correlation plots (with forced zero intercept) and Bland-Altman plots were used to investigate the differences between the biometric parameters from the right eye and left eye measurements for CCT, ACD, LT, AL, KEQ, WTW, and PD. For parameters where the differences Δ were normally distributed according to the Kolmogorow-Smirnov test for normality, the limits of agreement were derived as 1.96 times the population standard deviation [20]. For differences Δ that did not follow a normal distribution we used the reproducibility coefficient derived from 1.45 times the interquartile range as estimated from a nonparametric analysis [20].

For the vectorial measures (Purkinje I image (ICX and ICY), the pupil centre (PCX and PCY) and the keratometric astigmatism (KC0 and KC45)) we used polar scatterplots to display both vector components separately for left and right eyes (with direct and with mirror symmetry) and the differences between right and left eyes considering mirror symmetry. In addition, the 95% confidence ellipses derived from the variance-covariance matrices are shown in the scatterplots.

For testing the direct and mirror symmetry of the keratometer axis Axis1 for both eyes, the median of ΔAxis1D and ΔAxis1M was calculated. To test the likelihood that the observed similarities between the axes of fellow eyes under either direct or mirror symmetry could have arisen by chance, a resampling algorithm [1] (bootstrapping) was implemented where the axes Axis1_OS_ for the left eye were randomly sampled with replacement 1000 times and matched to the measurements of the right eye Axis1_OD_. Again, the axis differences ΔAxis1D and ΔAxis1M were analysed and the median of the 1000 bootstrap samples was recorded and compared to the median of ΔAxis1D and ΔAxis1M from the measurement of both eyes.

This analysis for the keratometric axis was performed for the entire dataset, and also for subgroups divided according to the axial length (short eyes (AL < 22 mm), medium eyes (22 mm ≤ AL ≤ 25 mm), long eyes (AL ≥ 25 mm)), mean corneal power (flat corneas (KEQ < 42 dioptres), medium corneas (42 dioptres ≤ KEQ ≤ 45 dioptres), steep corneas (KEQ ≥ 45 mm)), and corneal astigmatism (low astigmatism (KC < 0.25 dioptres), medium astigmatism (0.25 dioptres ≤ KC ≤ 2 dioptres), large astigmatism (KC ≥ 2 dioptres)).

For comparisons of the biometric parameters of both eyes the *t* test for paired samples was used for normally distributed data, and the Wilcoxon test was used for data which did not follow a normal distribution according to the Kolmogorov-Smirnov test for normality. Two-sided p-values less than 0.05 were considered statistically significant. The explorative nature of the investigation meant that it was not necessary to account for the issue of multiple statistical testing.

## Results

From the N = 38,720 biometric measurements transferred to us, and after applying the selection criteria, a dataset containing N = 26,840 measurements of eyes (13,420 patients with measurements from both eyes) was considered in our data analysis. The mean patient age was 69.58 ± 11.07 years (median 70.63 years, 95% confidence interval 41.31 to 87.39 years). Table 1 displays explorative data for the most relevant biometric measurements for the entire population and for the dataset split into left and right eyes. Table 2 lists the corresponding data for keratometry.

**Table 1:** Explorative data of the biometric measurements central corneal thickness (CCT), anterior chamber depth measured from corneal epithelium to the lens front apex (ACD), central lens thickness (LT), horizontal corneal diameter (WTW), pupil size (PD), the X and Y coordinates of the Purkinje I light reflex (ICX and ICY), and the X and Y coordinates of the pupil centre (PCX and PCY). The arithmetic mean (Mean), standard deviation (SD), median (Median) and the lower and upper boundary of the 95% confidence interval (2.5% quantile and 97.5% quantile) are provided for the entire dataset and also individually for the left and right eyes.

Data in mm		CCT	ACD	LT	AL	WTW	PD	ICX	ICY	PCX	PCY
All eyes (N = 26.840)	Mean	0.5516	3.1805	4.6310	23.9504	11.9294	4.0505	−0.0280	0.0794	−0.0290	−0.0270
	SD	0.0372	0.3988	0.4444	1.3429	0.4509	0.8921	0.4567	0.2213	0.3062	0.1637
	Median	0.5608	3.1797	4.6397	23.7973	11.9279	3.9761	−0.0382	0.0642	−0.0258	−0.0253
	2.5% quantile	0.4800	2.4109	3.7125	21.6729	11.0485	2.4842	−0.7370	−0.3203	0.5767	−0.3528
	97.5% quantile	0.6273	3.9680	5.4779	27.0880	12.8106	5.9935	0.6669	0.5791	0.5152	0.2935
Left eyes (N = 13420)	Mean	0.5517	3.1798	4.6364	23.9358	11.9389	3.9520	0.3936	0.0676	0.2168	−0.0467
	SD	0.0372	0.3963	0.4416	1.3432	0.4537	0.8646	0.1730	0.2203	0.1811	0.1609
	Median	0.5512	3.1793	4.6436	23.7834	11.9387	3.8697	0.3974	0.0538	0.2172	−0.0459
	2.5% quantile	0.4805	2.4151	3.7218	21.6541	11.0530	2.4290	0.0426	−0.3359	−0.1429	−0.3694
	97.5% quantile	0.6276	3.9570	5.4832	27.0451	12.8226	5.8645	0.7259	0.5542	0.5681	0.2662
Right eyes (N = 13420)	Mean	0.5514	3.1812	4.6255	23.9650	11.9200	4.1491	−0.4497	0.0912	−0.2749	−0.0073
	SD	0.0373	0.4013	0.4472	1.3425	0.4479	0.9081	0.1780	0.2217	0.1840	0.1641
	Median	0.5506	3.1799	4.6349	23.8068	11.9177	4.0917	−0.4493	0.0750	−0.2745	−0.0067
	2.5% quantile	0.4792	2.4083	3.7077	21.6945	11.0398	2.5414	−0.8024	−0.3071	−0.6371	−0.3331
	97.5% quantile	0.6270	3.9781	5.4695	27.1179	12.8026	6.0914	−0.1040	0.5931	0.0903	0.3142

**Table 2:** Explorative data of the biometric keratometry in terms of corneal radius in the flat (R1) and steep meridian (R2), the harmonic mean of the radii in both meridians (R), corneal power in the flat (K1) and steep meridian (K2), corneal astigmatism (KC), and the components of the 3D power vector with equivalent power (KEQ), and corneal astigmatism projected to the 0/90 degree (KC0) and the 45/135 degree (KC45) meridian. The arithmetic mean (Mean), standard deviation (SD), median (Median) and the lower and upper boundary of the 95% confidence interval (2.5% quantile and 97.5% quantile) are provided for the entire dataset and also individually for the left and right eyes.

		Data in mm			Data in dioptres					
		R1	R2	R	K1	K2	KC	KEQ	KC0	KC45
All eyes (N = 26.840)	Mean	7.7931	7.6189	7.7044	43.4735	44.3679	0.9945	43.8707	0.0444	0.0012
	SD	0.3061	0.3021	0.2970	1.6699	1.7412	0.7589	1.6647	0.5379	0.3203
	Median	7.7757	7.6066	7.6892	43.4068	44.3702	0.8094	43.8931	0.0272	0.0000
	2.5% quantile	7.2620	7.0733	7.1809	39.9931	40.9463	0.0978	40.5493	−0.9898	−0.6144
	97.5% quantile	8.4378	8.2421	8.3227	46.4822	47.7176	3.0116	47.0160	1.1951	0.6279
Left eyes (N = 13420)	Mean	7.7878	7.6137	7.6991	43.4146	44.3994	0.9943	43.9025	0.0524	0.0409
	SD	0.3066	0.3022	0.2972	1.6729	1.7437	0.7558	1.6679	0.5372	0.3137
	Median	7.7704	7.6019	7.6832	43.4367	44.3987	0.8116	43.9281	0.0357	0.0389
	2.5% quantile	7.2574	7.0670	7.1794	40.0133	40.9744	0.0963	40.5770	−0.9762	−0.5566
	97.5% quantile	8.4338	8.2369	8.3156	46.5060	47.7634	3.0090	47.0277	1.2112	0.6436
Right eyes (N = 13420)	Mean	7.7985	7.6240	7.7098	43.3423	44.3365	0.9946	43.8390	0.0364	−0.0385
	SD	0.3055	0.3020	0.2968	1.6664	1.7381	0.7620	1.6610	0.5384	0.3218
	Median	7.7807	7.6114	7.6952	43.3784	44.3411	0.8075	43.8585	0.0207	−0.0431
	2.5% quantile	7.2674	7.0793	7.1820	39.9723	40.9035	0.0984	40.5328	−1.0068	−0.6488
	97.5% quantile	8.4414	8.2598	0.3263	46.4413	47.6739	3.0173	47.0033	1.1828	0.6098

**Fig 1:**
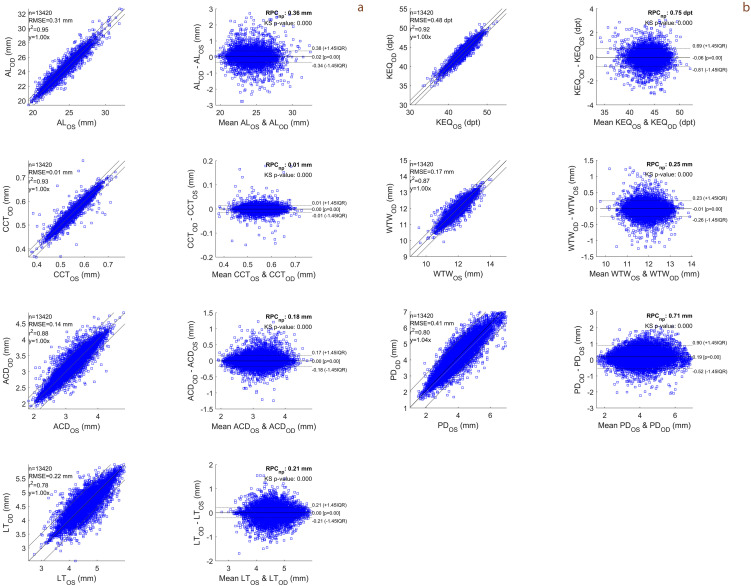
Correlation plots (left graphs) and Bland-Altman plots (right graphs) showing the correlations and the differences between the biometric parameters for the right and the left eyes of individuals. Panel a displays the axial length AL, central corneal thickness CCT, anterior chamber depth measured from corneal epithelium to the lens front apex, and the central lens thickness LT. Panel b shows the keratometric equivalent power KEQ, the horizontal corneal diameter WTW and the pupil size PD. The correlation plots also show the linear regression line (with enforced zero intercept, slope mentioned in the legend), the squared correlation coefficient r^2^ (i.e., the coefficient of determination) and the root mean squared fit error RMSE. In the Bland-Altman plot the difference between both eyes is plotted against the mean of both eyes. Since none of the differences were normally distributed (Kolmogorov-Smirnov KS p value shown in the legend) we used the ± 1.45· interquartile range interval as a nonparametric estimate for the limits of agreement.

Fig 1 shows correlation plots on the left side and Bland-Altman plots on the right side, with individual plots in each case for the parameters axial length, central corneal thickness, anterior chamber depth and central lens thickness (Fig 1a) and for the keratometric equivalent power, the horizontal corneal diameter and the pupil size (Fig 1b). The linear regression lines in the correlation plots were calculated on the basis of a forced zero intercept. The values of the root mean squared fit error (RMSE), the squared correlation coefficient(r², the coefficient of determination) and the slope of the regression line are shown in the text annotations on the graphs. In generating the Bland-Altman plots we tested the differences between the biometric measures from the right and left eye for normality (P value according to Kolmogorov-Smirnov (KS p-value). Since none of the differences shown in the plots followed a normal distribution, an estimate of the reproducibility coefficient based on the interquartile range (nonparametric statistics) is shown, together with the mean difference and the ± 1.45 interquartile range, instead of the limits of agreement in the graphs.

**Fig 2:**
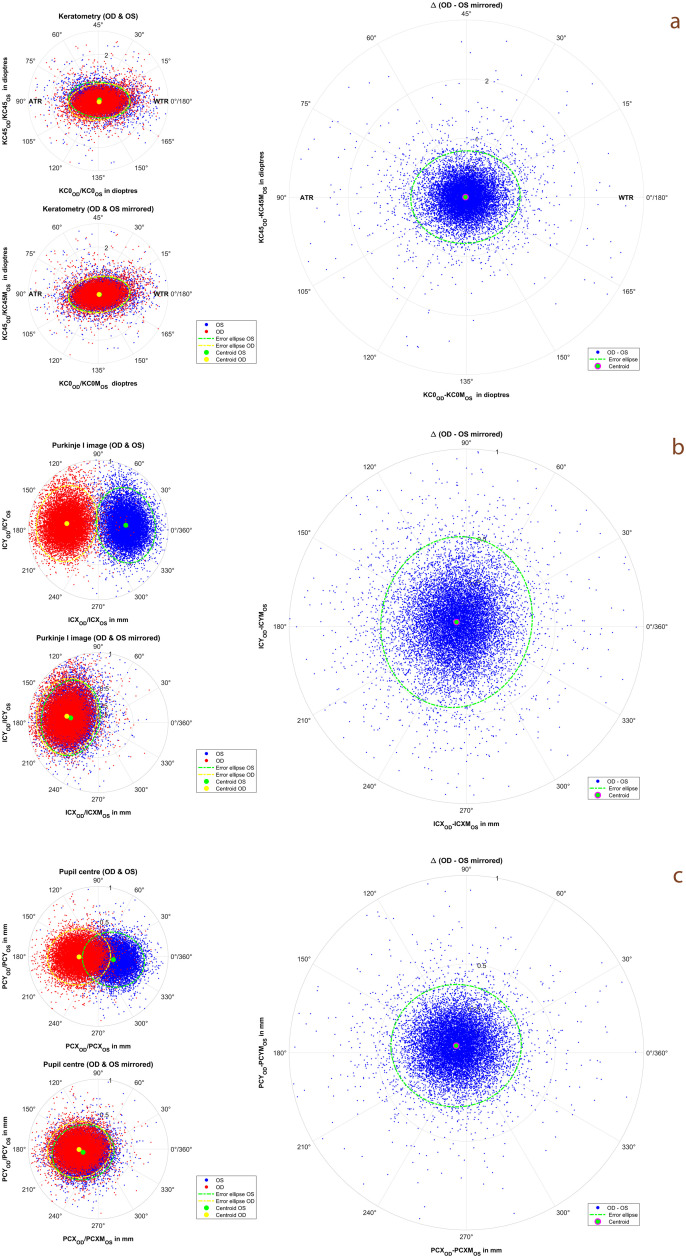
Polar scatterplot of the vector components of keratometric astigmatism (a), the coordinates of the Purkinje I image (b), and the location of the pupil centre (c). In the upper left graphs the vector components for the left (OS) and right (OD) eye are plotted. The lower left graphs display the situation when the left eye is mirrored with respect to the vertical axis (by flipping the sign of the KC45, the ICX, or the PCX vector component). In the polar scatterplots on the right graphs the difference between the right and the mirrored left eye is shown. For all scatterplots we included centroids and the 95% confidence ellipses derived from the variance-covariance matrix of the vectorial data. WTR and ATR in panel a refer to the orientation of astigmatism with-the-rule (flat keratometer axis between 0 and 30 degrees or between 150 and 180 degrees) and against-the-rule (flat keratometer axis between 60 and 120 degrees) respectively.

Fig 2 uses polar scatterplots to show the vector components for the astigmatic power vector components KC0 and KC45 from keratometry (Fig 2a), for the X and Y coordinates of the Purkinje I image ICX and ICY (Fig 2b), and for the X and Y coordinates of the pupil centre PCX and PCY (Fig 2c). The upper left graphs display the two vector components against each other (KC0, ICX, or PCX at the X and KC45, ICY, or PCY at the Y axis), whereas in the lower left graphs, all left eyes were mirrored with respect to the vertical plane (i.e., KC45, ICX, and PCX were flipped in sign for left eyes). The graphs on the right show the differences in the vector components (subtracting the mirrored left eye components from the respective right eye components). For all polar scatterplots the centroid and the 95% confidence ellipses are provided for left and right eyes (left graphs) and for the differences (right graphs).

Tables 3 and 4 display the descriptive data for the parameter differences (right eye data minus the corresponding left eye data). As before, for the three vectorial parameters (corneal astigmatism, location of the Purkinje I image and location of the pupil centre) the data for the differences are also shown for the situation where the data for the left eyes were mirrored about the vertical plane.

**Table 3:** Differences (Δ) between the right eye and the left eye biometric measures in terms of arithmetic mean (Mean), standard deviation (SD), median (Median) and the lower and upper boundary of the 95% confidence interval (2.5% and 97.5% quantile). Biometric measures include central corneal thickness CCT, anterior chamber depth measured from the corneal epithelium to the lens front apex, central lens thickness LT, axial length AL, horizontal corneal diameter WTW, pupil size PD, and the X and Y coordinates of the Purkinje I image location ICX and ICY. Differences between right and left eyes are all statistically significant (P < 0.001) but below clinical relevance.

N = 13420	Biometric distances in mm					
	ΔCCT	ΔACD	ΔLT	ΔAL	ΔWTW	ΔPD
Mean	−0.0003	0.0014	0.0018	0.0292	−0.0189	0.1972
SD	0.0101	0.1420	0.1418	0.3116	0.1665	0.4067
Median	−0.0005	−0.0036	−0.0037	0.0218	−0.0128	0.1902
2.5% quantile	−0.0176	−0.2680	−0.2678	−0.5838	−0.3609	−0.6026
97.5% quantile	0.0172	0.3130	0.3129	0.6467	0.2942	1.0303

**Table 4:** Differences (Δ) between the right eye and the left eye biometric measures in terms of arithmetic mean (Mean), standard deviation (SD), median (Median) and the lower and upper boundary of the 95% confidence interval (2.5% and 97.5% quantile). Data measures include the X and Y coordinates of the Purkinje I image location ICX and ICY, the X and Y coordinates of the pupil centre PCX and PCY, corneal astigmatism defined as the difference of power between the steep and flat meridian KC, and the 3 keratometric power vector components with equivalent power KEQ, and the corneal astigmatism projected to the 0/90 degree KC0 and the 45/135 degree meridian KC45. For the 3 vector measures (Purkinje I image, pupil centre, and corneal power) the differences where left eyes are mirrored to right eyes (indicated by (.)_M_) with respect to the vertical axis (by flipping the sign of PCX and KC45) are also provided. The statistical significances (Pvalues) indicate that the differences between right and left eyes are statistically significant except for KC and KC45_M_.

N = 13420	Purkinje I image in mm			Pupil centre in mm			Keratometry vector components in dioptres				
	ΔICX	ΔICX_M_	ΔICY	ΔPCX	ΔPCX_M_	ΔPCY	ΔKC	ΔKEQ	ΔKC0	ΔKC45	ΔKC45_M_
Mean	−0.8433	−0.0560	0.0235	−0.4917	−0.0581	0.0394	0.0003	−0.0635	−0.0160	−0.0794	0.0024
SD	0.3045	0.1746	0.1968	0.3228	0.1501	0.1409	0.6007	0.4706	0.3780	0.5494	0.3197
Median	−0.8439	−0.0506	0.0237	−0.4923	−0.0550	0.0405	−0.0058	−0.0623	−0.0136	−0.0865	−0.0002
2.5% quantile	−1.4348	−0.4368	−0.3754	−1.1430	−0.3609	−0.2361	−1.2481	−0.9919	−0.7819	−1.1519	−0.6145
97.5% quantile	−0.2489	0.25778	0.4247	0.1662	0.2289	0.3180	1.2485	0.8689	0.7172	1.0087	0.6526
P value	<0.001	<0.001	<0.001	<0.001	<0.001	<0.001	0.6769	<0.001	<0.001	<0.001	0.8019

Table 5 lists the data for the median differences of the keratometric astigmatism axes where either direct symmetry (ΔAxis1D(or mirrored symmetry (ΔAxis1M) is considered, together with the results of permutations (1000 random samples with replacement) in the axes of the left eyes. The median differences are shown for the entire dataset and for each of the subgroups axial length, keratometric equivalent power, and keratometric astigmatism. ΔAxis1D without permutations shows significantly lower values compared to ΔAxis1D with permutations for the entire population and for all subgroups except for the group of low corneal astigmatism (KC < 0.25 dioptres), and ΔAxis1M without permutations shows significantly lower values compared to ΔAxis1M with permutations for the entire population and all subgroups.

**Table 5:** Median difference of the keratometric astigmatism axis (right eye minus left eye) considering direct symmetry ΔAxis1D and considering mirror symmetry Δaxis1_M_ for the entire dataset and subgroups for axial length, corneal equivalent power, and corneal astigmatism. In columns 3 and 4 the data are shown for the keratometric axes measured for both eyes, and in columns 5 and 6 the data are shown for the situation where measured data for the right eye are matched to randomly permuted data for the left eye measurements.

N = 13.420, data in degree		Measured keratometric axes from left and right eye		With permutation of axes from left eye within the patient group	
		ΔAxis1D	ΔAxis1M	ΔAxis1D	ΔAxis1M
Entire dataset		29.04	14.95	45.39	44.27
Grouped for axial length AL	AL < 22 mm (N = 629)	29.49	14.92	44.70	44.20
	22 ≤ AL ≤ 25 mm (N = 10,308)	29.05	15.06	45.68	44.31
	AL > 25 mm (N = 2,483)	28.71	14.57	41.61	41.24
Grouped for corneal equivalent power KEQ	KEQ < 42 dioptres (N = 1,515)	30.35	16.70	45.53	44.56
	42 ≤ KEQ ≤ 45 dioptres (N = 8,654)	28.45	14.90	45.46	44.40
	KEQ > 45 dioptres (N = 3,251)	29.80	14.38	44.27	43.02
Grouped for corneal astigmatism KC	KC < 0.25 dioptres (N = 482)	48.25	31.87	45.13	44.94
	9,25 ≤ KC ≤ 2.0 dioptres (N = 11,904)	29.70	15.51	45.56	44.24
	KC > 2.0 dioptres (N = 1,034)	16.34	8.04	40.40	40.28

**Fig 3:**
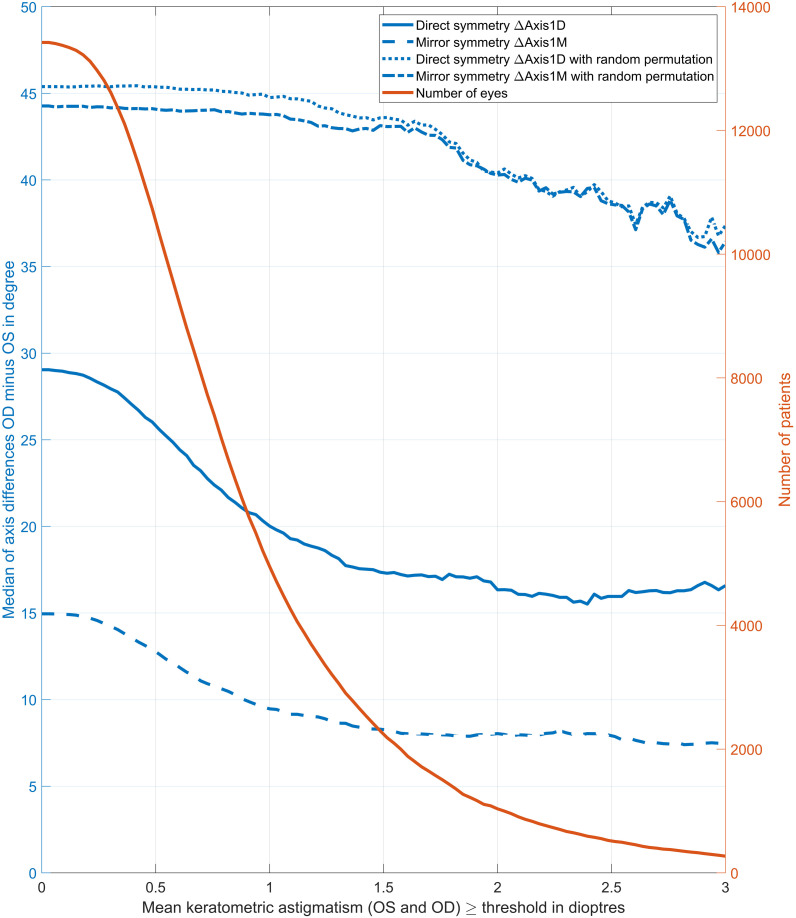
Direct and mirror symmetry of the keratometry axis as a function of a lower threshold for the mean keratometric astigmatism. The solid blue line refers to the median deviation between the keratometer axes of both eyes, and the dashed blue line to the median deviation between the keratometer axes after mirroring of the left eye with respect to the vertical axis. The dotted and dashdotted lines show the reference when the median deviations are derived from the right eye measurement and a random permutation (1000 times sampling with replacement) from the left eye measurements without mirroring of left eyes (dotted line) or with mirroring of left eyes (dash-dotted line). The graph indicates that, especially when small cylinder values are excluded from the analysis, mirror symmetry outperforms direct symmetry which again outperforms a random match of both keratometer axes of both eyes.

Fig 3 shows ΔAxis1D and ΔAxis1M with and without permutation of the left eye keratometric axis measures as a function of the threshold for keratometric astigmatism. The red line gives the number of eyes with a keratometric astigmatism larger than the threshold (axis label to the right). When all data are included (left margin of the plot) the axes of the left and right eyes show a median deviation from mirror symmetry of around 15 degrees and a median deviation from direct symmetry of around 28 degrees, whereas a match to random axes shows a median deviation of around 45 degrees. When lower astigmatism values are excluded (according to the threshold shown on the X axis of the graph) the performance of both direct symmetry and mirror symmetry increases systematically (e.g., for KC > 2.0 dioptres to 16 and 8 degrees) whereas the ΔAxis1D and ΔAxis1M with random permutations decrease only slightly to around 37 degrees.

## Discussion

The adoption of optical biometry has significantly improved the predictability of the refractive outcome after cataract surgery [1,2]. However, in some cases optical biometry of one eye is not possible or is not available prior to cataract surgery, and the biometric measures for IOL power calculation have to be estimated. If it could be established that both eyes of an individual show similar biometric measures in most cases, the easiest way would be to use the biometry of the fellow eye for IOL power calculation. However, the similarity of biometric measures of both eyes has a wider relevance and potential application than situations where biometry of both eyes is not available. For instance comparison of the measures could also help to understand effects such as retinal image size disparities [17] or aniseikonia [16] where biometric measures of fellow eyes differ.

In the case of scalar biometric measures such as AL or ACD the concept of inter-eye similarity is quite simple, and the similarity of both eyes can be tested by directly considering the difference between the two eyes of a patient [4,5,8,9,15]. However, when dealing with vectorial measures such as keratometry or the location of the Purkinje I reflex or the pupil centre, a direct comparison of the vector components might not reflect symmetry conditions in general [9,10]. As an example, for the location of the pupil centre, the decentred location of the fovea means that the visual axis is somewhat tilted, even if we assume that all refractive components of the eye are coaxially aligned to an ‘optical axis’. Since the location of the fovea in left and right eyes is typically mirrored with respect to the vertical plane, the visual axes of both eyes also show some mirror symmetry and we would consequently expect the entrance pupil locations of both eyes to be quite similar under mirror symmetry. In this context mirror symmetry means that the Y components are similar and the X components are similar in the absolute value but with inverted sign. For the location of the Purkinje I reflex we would expect something similar since, as with the pupil centre location, it is also affected by the tilt of the visual axis.

When considering the 3D power vector of keratometry we have to differentiate between the equivalent power KEQ and the two astigmatic components. The keratometric equivalent power could be processed easily as a scalar measure with a direct comparison of the fellow eye measures. However, clinicians are used to dealing with keratometric astigmatism in terms of absolute values (keratometric astigmatism KC and, e.g., the axis of the flat (or steep) meridian). Since the keratometric axis shows a periodicity of 180 degrees, a direct symmetry would mean that KC and the flat (and steep) axis values Axis1 of both eyes would match [9,11–13]]. However, if we assume, as with the location of the Purkinje image or the pupil centre, that fellow eyes of a patient show mirror symmetry with respect to the vertical plane, then KC of both eyes should be similar but the keratometer axis of one eye should be similar to 180 degrees minus the keratometer axis of the fellow eye [10]. When converted to power vector components, this means that the KC0 components of both eyes are similar and the KC45 components show similar absolute values but are inverted in sign [3].

In the present paper we used a large biometric dataset from a modern optical biometer of measurements made on a patient cohort before cataract surgery to analyse the symmetry of biometric measures of both eyes. For that purpose we filtered the dataset to have strictly paired measurements taken on the same examination day from both eyes of each subject. After an explorative evaluation of the data we used correlation plots and Bland-Altman plots (Fig 1) to investigate the similarity of the scalar measures from both eyes of the study population. The graphs show that, for example, axial length (r^2^ = 0.95), central corneal thickness (r^2^ = 0.93) and keratometric equivalent power (r^2^ = 0.92) show an excellent correlation, whereas the central lens thickness (r^2^ = 0.78) or the pupil size (r^2^ = 0.80) of both eyes show much more scatter. Except for the pupil size where the slope of the regression line (with enforced zero intercept) was 1.04 indicating a systematically larger pupil in the right compared to the left eye by around 4%, the slopes of the regression lines for all other scalar measures were 1.00. However, the Bland-Altman plots show that the biometric measures could show large differences between left and right eyes, even with good to excellent correlation: for example, we observed differences in axial length of up to 3 mm (reproducibility coefficient 0.36 mm) or in the keratometric equivalent power of up to 3.5 dioptres (reproducibility coefficient 0.75 dioptres). Because none of the differences of the scalar biometric measures between both eyes were normally distributed, non-parametric statistics were required to extract the characteristic metrics in the Bland-Altman plots [20]. Tables 3 and 4 list the descriptive data of the differences of the right eye measures minus the left eye measures for all scalar measures and for the components of the vectorial measures. To give a better insight, the differences in the components of the vectorial measures when left eye values are mirrored to right eyes with respect to the vertical axis are also provided. From the mean and median values we see that the mean/median differences for the vector components with mirroring left eyes is systematically lower than the mean/median differences in a direct comparison without mirroring left eyes (−0.06/-0.05 mm versus −0.84/-0.84 mm for ICX, −0.06/-0.06 mm versus −0.49/-0.49 mm for PCX, and 0.00/0.00 dioptres versus −0.08/-0.09 dioptres for KC45). This comparison supports the assumptions that all 3 vectorial measures (location of the Purkinje I image, location of the pupil centre, and keratometric astigmatism) exhibit mirror symmetry with respect to the vertical axis rather than direct symmetry.

The polar scatterplots in Fig 2 show the situation for the vector components of the keratometric astigmatism (KC0 in X and KC45 in Y direction, Fig 2a) in a double angle plot, and for the Cartesian coordinates of the Purkinje I image (ICX in X and ICY in Y direction, Fig 2b), and for the Cartesian coordinates of the pupil centre (PCX in X and PCY in Y direction, Fig 2c). The upper left graph in Fig 2a shows much more scatter in the X than in the Y direction for both left and right eyes, indicating that keratometric astigmatism is more pronounced with-the-rule or against-the-rule than in the oblique axes. This is also reflected by the horizontal orientation of the 95% error ellipses. However, we also see that, even though the centroids overlap, the error ellipses for left eyes and right eyes do not perfectly match. The error ellipse for left eyes (green dashed line) is somewhat tilted from the horizontal orientation clockwise, whereas the error ellipse for the right eyes (yellow dashed line) is somewhat tilted anti-clockwise. This symmetrical tilt between the error ellipses suggests that there might be mirror symmetry in the keratometric astigmatism with respect to the vertical axis (non-mirrored KC0 and mirrored KC45 components). In the lower left graph the sign of the KC45 component for left eyes has been reversed, and as a consequence the ellipses for the (mirrored) left and the right eyes match properly. We therefore decided to plot the corresponding vector differences between the right eye and the mirrored left eye on the right graph. As can be seen from this graph, there is no systematic offset (centroid close to zero), the scatter is much smaller compared to the scatter in the left graphs, and the scatter is more symmetric indicated by a roundish shape of the error ellipse. However, even though most of the datapoints are concentrated close to the centre, there are also some rare datapoints where the magnitude of the vector difference between keratometric astigmatism of both eyes is up to 3 dioptres (radial distance to the centre of the polar plot). From the upper left graph in Fig 2b it is clear that the location of the Purkinje I image shows some mirror symmetry with respect to the vertical axis since the X coordinates of left and right eyes are similar in absolute value and inverted in sign. To test for mirror symmetry the lower left graph shows the situation where the X coordinate has been inverted in sign for left eyes. Even though the scatterplot shows a large scatter, most pronounced in the Y direction as indicated by the vertical orientation of the 95% error ellipse, the centroids show a large offset from zero mostly in the temporal direction and slightly upwards (X/Y coordinates are −0.39/0.07 mm for mirrored left eyes and −0.45/0.09 mm for right eyes). Together with the slight disparities in both error ellipses, the disparities in the centroids indicate that the Purkinje I reflex does not fully show mirror symmetry between left and right eyes. This discrepancy is also underlined by the right graph in which the centroid is slightly shifted from the origin towards the upper left and the error ellipse shows some asymmetry. Again, from the upper left graph in Fig 2c it is clear that the location of the pupil centre shows some mirror symmetry with respect to the vertical axis in that the X coordinates of left and right eyes are similar in the absolute value and inverted in sign. To test for mirror symmetry the lower left graph shows the situation where the X coordinate has been inverted in sign for left eyes. Even though the scatterplot shows a mostly symmetric scatter according to the roundish shape of the 95% error ellipse, the centroids show some offset from zero, mostly in the temporal direction (X/Y coordinates are −0.22/-0.05 mm for mirrored left eyes and −0.27/-0.01 mm for right eyes). The slight disparities in the centroids indicate that the pupil centre location does not fully exhibit mirror symmetry between left and right eyes. This discrepancy is also evident in the right graph which shows that the centroid is slightly shifted from the origin towards the upper left even though the scatter seems to be symmetric (indicated by a roundish shape of the 95% error ellipse).

Since clinicians mostly deal with keratometric astigmatism expressed in absolute value and axis, the concept of analysing direct and mirror symmetry was transferred to the keratometric axis. Considering the 180-degree periodicity of the keratometric axis, we calculated the deviation of the keratometric axis of both eyes based on the assumptions of both direct symmetry and mirror symmetry. For the entire dataset we obtain values for the median ΔAxis1D = 29.04 degrees and for ΔAxis1M = 14.95 degrees. For reference we implemented a bootstrapping concept where the axes of the left eyes were randomly sampled 1000 times [10]. The match between the measurement of the right eye and the random permutation for the left eye gives an indication of the axis deviation values ΔAxis1D and ΔAxis1M for a random match of both eyes maintaining the probability distribution function for Axis1_OS_ and Axis1_OD_. As shown in Table 5 the corresponding reference values for the entire dataset are ΔAxis1D = 45.39 degrees and ΔAxis1M = 44.27 degrees.

For a better understanding of the influencing parameters, we subdivided the dataset into subgroups according to axial length, keratometric equivalent power and keratometric astigmatism. With the exception of the groups of keratometric astigmatism, where we observe large median values in ΔAxis1M = 31.87 degrees for KC < 0.25 dioptres and small median values ΔAxis1M = 8.04 degrees (and ΔAxis1D = 16.34 degrees) for KC > 2.0 and ΔAxis1M, the differences in the 4 metrics are quite low. This underlines the common knowledge in ophthalmology that the orientation of the axis is undefined for very low values of keratometric astigmatism. To investigate this effect of random variation of the axis for low values of keratometric astigmatism we defined and varied a lower threshold for mean KC (left and right eye) and plotted the median variation of the keratometer axis ΔAxis1D and ΔAxis1M as a function of this threshold. From Fig 3 we see that when low keratometric astigmatism values are excluded from the analysis, the median difference in the keratometer axis of both eyes ΔAxis1D / ΔAxis1M decays from around 29/15 degrees to around 20/9 degrees for KC values ≥ 1 dioptre, or 17/8 degrees for KC values ≥ 2 dioptres. This is in accordance to the study of Guggenheim et al. [10], who found a median ΔAxis1D / ΔAxis1M of 20/10 degrees for KC values ≥ 1 dioptre.

However, our study has some limitations: firstly, our results are based on a single-centre dataset with biometric measurements derived from the LenStar before cataract surgery. With other biometers or other study populations the results might differ to some extent. Secondly, we analysed the vectorial data only under assumptions of direct symmetry and mirror symmetry with respect to the vertical axis. Even though we feel that these two symmetry conditions may be the most relevant ones, there might be other symmetry conditions which better match our data. And finally, we did not systematically study all of the predictors for similarity in the scalar measures and for direct or mirror symmetry in the vector measures: for example, we did not split our dataset into subsets for keratometric astigmatism with-the-rule, against-the-rule-or oblique astigmatism.

In **conclusion**, our study is based on a large dataset from one study centre with paired biometric measures from both eyes made before cataract surgery using the Haag-Streit LenStar 900 optical biometer. We found that all scalar measures of both eyes of an individual show a high degree of agreement, but with large differences between the two eyes in some rare cases. For the vector measures such as the keratometric astigmatism and the location of the Purkinje I reflex and pupil centre we were able to show that there is mostly mirror symmetry with respect to the vertical axis rather than direct symmetry. This information about similarity and symmetry between left and right eyes could be used to calculate the lens power using the corresponding biometric parameters from the fellow eye in cases where a biometric measurement cannot be performed one of the eyes, or where the data for one eye are unavailable. The information that there are only rare cases with larger disparities between the eyes suggests that biometric measures of the opposite eye could be used for a double-check of biometric measures in a clinical routine process for lens power calculation prior to cataract surgery.
